# Association between smartphone usage and health outcomes of adolescents: A propensity analysis using the Korea youth risk behavior survey

**DOI:** 10.1371/journal.pone.0294553

**Published:** 2023-12-06

**Authors:** Jong Ho Cha, Young-Jin Choi, Soorack Ryu, Jin-Hwa Moon

**Affiliations:** 1 Department of Pediatrics, Hanyang University Medical Center, Seoul, Korea; 2 Department of Pediatrics, Hanyang University Guri Hospital, Guri, Korea; 3 Biostatistical Consulting and Research Lab, Medical Research Collaborating Center, Hanyang University, Seoul, Korea; 4 Division of Pediatric Neurology, Department of Pediatrics, Hanyang University College of Medicine, Seoul, Korea; West University of Timisoara: Universitatea de Vest din Timisoara, ROMANIA

## Abstract

**Objectives:**

We aimed to investigate the association between smartphone use and adverse behavioral health outcomes using nationwide Korea Youth Risk Behavior Web-based Survey data for 2017 and 2020.

**Methods:**

The 2020 data (N = 54,809) were used to analyze the relationships between daily smartphone usage time (non-user, 0–2 h [hour], 2–4 h, 4–6 h, 6–8 h, and > 8 h), and adverse health outcomes (stress, sleep, depression, suicide, substance use, and smartphone overdependence). A 1:1 propensity score matching (PSM) was used to control for confounding variables.

**Results:**

A total of 40,998 adolescents with < 4 h/day and > 4 h/day of usage were included. Adolescents’ mean smartphone usage time in 2020 increased compared to that in 2017 (weighted % of > 2 h/day; 64.3% vs. 85.7%). The curvilinear relationships between smartphone usage time and adverse health outcomes were prominent after > 4 h/day. Adolescents using smartphones 2–4 h/day showed no increased adverse health outcomes compared to non-users, except for smartphone overdependence. Using a smartphone > 4 h/day was significantly associated with stress perception (1.16; 1.11–1.22), suicidal ideation (1.22; 1.13–1.31), and substance use (alcohol, 1.66; 1.57–1.75) after PSM.

**Conclusions:**

Our study demonstrated the curvilinear relationship between smartphone usage time and adverse health outcomes in adolescents. Our findings can help establish smartphone usage guidelines for adolescents.

## Introduction

Smartphones have become essential platforms in the lives of young people. Adolescents’ daily lives are connected to smartphones for various purposes, and this trend has been accelerated by school closures and social distancing due to the coronavirus disease 2019 (COVID-19) outbreak [1]. According to a recent study on adolescents’ data from the Korean Children and Youth Panel Survey, time spent on smartphones has surged significantly, with the largest increase in watching videos, such as on YouTube [2].

As smartphone usage time increases, growing evidence suggests that the smartphone is related to many adverse health effects among adolescents, including sleep difficulties [3–5], ophthalmologic disorders [6], and musculoskeletal disorders [7]. In addition, the literature has shown that smartphone usage by adolescents is likely to be associated with psychopathologies and psychiatric disorders [8–10].

In 2013, the American Academy of Pediatrics (AAP) made a statement that recommended limiting screen time up to 2 hours a day; this remains a universally recommended time limit regarding digital media use in pediatric populations [11]. Although the policy statement included both children and adolescents, it is not clear whether the guideline considers adolescents, who are more receptive to new technologies than younger children. The proportion of adolescents and young adults using a smartphone for more than 3 hours a day was 50% in Switzerland [12], 43% in the United States of America (USA) [9], and 44% in South Korea [13]. Therefore, there is a need for re-evaluating digital media usage time recommendations for adolescents by accommodating both the increasing trend of smartphone usage and its influence on adverse health issues.

According to Bianchi and Philips [14], “problematic smartphone use (PSU)” has been introduced as a behavioral addiction with characteristics resembling other addictive behaviors in 2005. On the other hand, Billieux et al. [15] showed that PSU is a heterogeneous and multifactorial condition rather than an addictive behavior. Meanwhile, a growing body of evidence suggests the relationship between digital media usage and poor health outcomes is not proportional; rather a favorable relationship is manifested to some extent. These studies reported that adolescents who used the Internet for less than 2 to 2.5 hours a day had better perceived physical and mental health conditions [16,17]. Similar results have been reported for smartphones, in the Korea Youth Risk Behavior Web-based Survey (KYRBWS) conducted in 2017, where suicidal risk was significantly lower in adolescents who reported using a smartphone 1–2 hours a day [13]. Przybylski et al. reported that the digital media usage is not solely harmful [18,19]. Moreover, they showed that the relationship between technology engagement and mental health is changing dynamically, with the association with depression becoming less prominent; however, the association between social media use and emotional problems has become more prominent over time [20]. Therefore, the link between smartphone use and mental health needs to be re-discussed in light of the current heightened usage and the traits of the current adolescents who are familiar with digital media devices.

Herein, we hypothesized that there may be changes in smart device usage time by year during adolescence and that an increase in smart device usage time may have varying effects on adverse health outcomes. Using nationwide data for South Korea in 2017 and 2020, we aimed to evaluate the smartphone usage time of the adolescent population and investigate the association between smartphone overuse and adverse health outcomes using propensity score matching (PSM).

## Materials and methods

### Data source and study population

**Fig 1:**
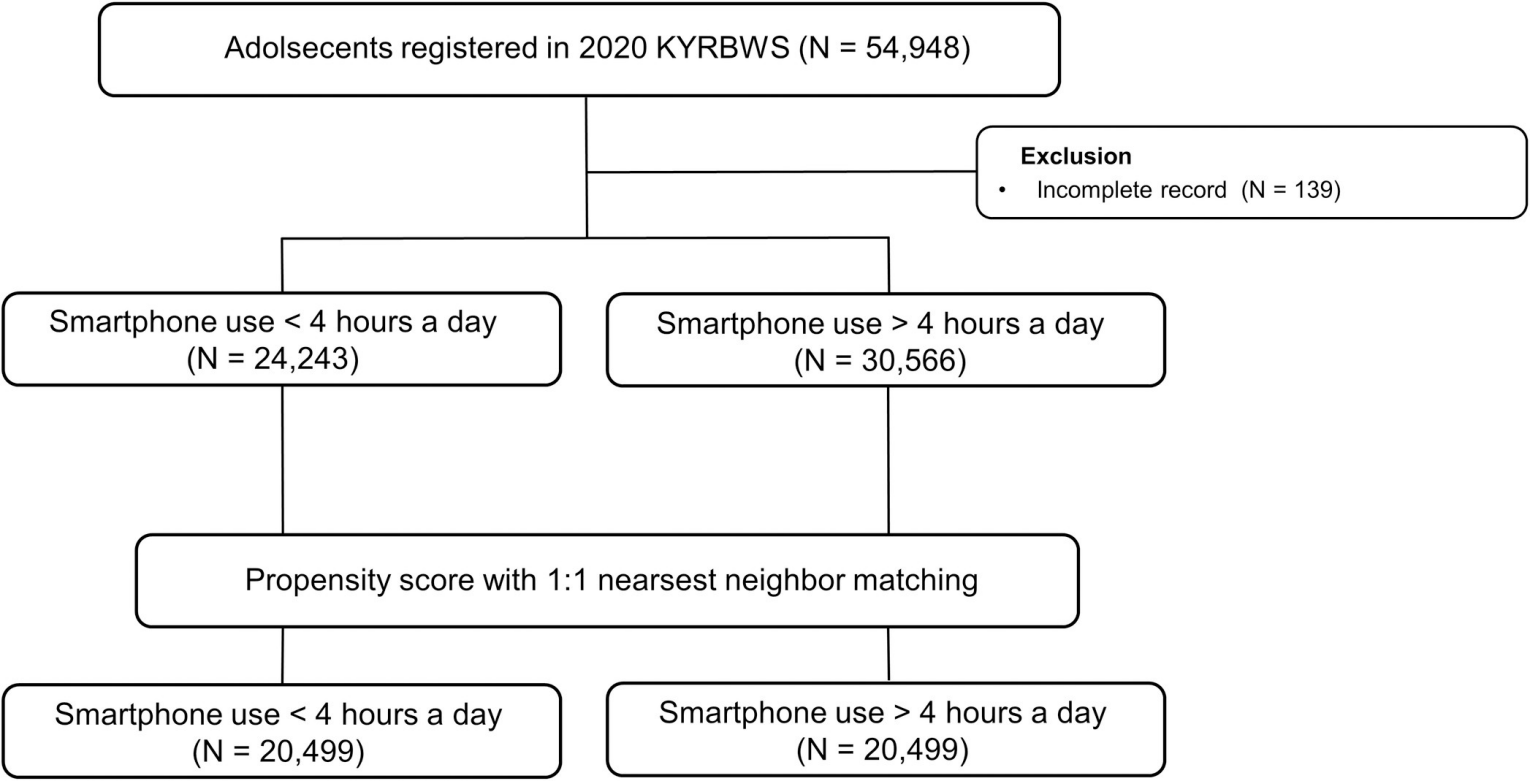
Study population flow chart used in the study. Abbreviations: KYRBWS, Korea Youth Risk Behavior Web-based Survey.

We used the data of the KYRBWS conducted by the Korea Centers for Disease Control and Prevention in 2017 and 2020. KYRBWS is a nationwide, annual school-based survey that investigates health risk behaviors using self-administered questionnaires. It has a complex sampling design with stratification, clustering, and multistage sampling to obtain a nationally representative sample of adolescents in South Korea [21]. The survey comprises 103 questions divided into 15 categories regarding health-related physical and mental health. A questionnaire regarding smartphone usage was conducted in 2017 for the first time and re-conducted in 2020. In 2020, 54,948 students from 793 middle and high schools were randomly selected and anonymously completed the self-administered questionnaire. Fig 1 presents the study design flow from enrollment to PSM analysis. We excluded adolescents without baseline demographic information (N = 139).

### Ethics statement

The Korea Centers for Disease Control and Prevention sampled nationwide subjects through the web-site and the participants agreed with a simple consent before the survey. The use of the raw data from the KYRBWS adheres to the personal information protection and statistics law. The survey provides the data without any personally identifiable information. The study was approved by Hanyang University Guri Hospital (IRB No. GURI 2022-01-027).

### Smartphone usage time

Questions on usage time of smartphones were obtained using a self-answered questionnaire including a dichotomous question: “Have you used your own or someone else’s smartphone in the last 7 days?” and a short-answer question: “In the last 7 days, how many hours per day did you use the smartphone in average?”. Participants’ responses were listed in 10-minute increments (i.e., 30 min, 1 hour, 1 hour 40 min, etc.). The daily average usage time of smartphones was measured using the following formula: (weekday average smartphone usage time * 5 + weekend average smartphone usage time * 2)/7. We considered both weekday and weekend usage time to reflect usage behavior on both school days and holidays. Smartphone overdependence was measured based on a self-answered questionnaire developed by the National Information Society Agency in 2016 [22]. It consists of 10 items rated on a four-point Likert scale, ranging from “strongly disagree,” coded 1, to “strongly agree,” coded 4. In the present study, the scores for this scale ranged from 0 to 40. Cut-off values of 23 and 31 were suggested for the potent risk group and high-risk group, respectively. Adolescents scoring more than 31 points in the questionnaire were defined as having smartphone overdependence. The Cronbach’s alpha coefficient was 0.91 [23].

### Measured health outcome variables

The selected health outcome variables were categorized as follows: (1) mental health, (2) substance use, and (3) obesity. We selected stress perception, dissatisfaction with sleep, depressive symptoms, suicidal ideation, suicidal plan, and suicide attempts as outcome variables regarding mental health. The survey questions regarding depressive symptoms assessed subjective feelings of hopelessness, which could be serve as a proxy measure. Suicidality was assessed by asking the participants whether they had suicidal ideas, plans, or attempts in the past 12 months, which could be part of their current presentation [24]. Recent spending on alcohol and smoking was selected as variables regarding substance use. Lastly, obesity was defined as the participant’s body mass index exceeding 25 kg/m^2^. The KYRBWS questionnaires used in the study are summarized in S1 Table. Each variable is composed of several Likert scales and transformed into dichotomous scales.

### PSM

We used PSM to control for potential confounding factors that could impact the health outcomes of study populations. We created matched sets of adolescents between smartphone usage of > 4 hours a day and < 4 hours a day. We set 4 hours as a cut-off point owing to the following reasons. First, the literature has shown that poor health outcomes appear when usage time exceeds 3–4 hours a day [17,19]. Second, 4 hours of daily usage time was the inflection point in the association between health-related outcomes in our study. Selected confounders for propensity score estimation were age, sex, residence, socioeconomic status, and academic achievement.

Demographic information included in the PSM was obtained from the KYRBWS data. For complex sampling, cities including Seoul and metropolitan areas (e.g., over one million people) were classified as large cities and cities with a population of less than 100,000 were classified as small cities. Each individual was asked about his/her subjective socioeconomic status and academic achievements on a five-point Likert scale, and those variables were categorized into three levels (i.e., upper and upper-middle to high, middle to middle, upper-lower and lower to low).

Then, the nearest available matching based on the estimated propensity score was performed with a 1:1 ratio. The balance between the two groups was assessed on selected covariates and evaluated using the standardized mean difference (SMD). Sampling weights with regional strata were also added in the matching process to minimize bias in parameter estimates.

### Statistical analysis

We compared smartphone usage time between the 2017 and 2020 surveys using chi-square analysis. The baseline demographic characteristics between the two groups were calculated with weighted percentages and compared with the SMD. Before PSM, the study population was stratified by average smartphone usage time for every 2 hour (non-user, 0–2 hours, 2–4 hours, 4–6 hours, 6–8 hours, and > 8 hours). Then, a univariable logistic regression analysis was used to calculate the OR with 95% C.I. of each usage group with the non-user group as a reference. After PSM, a univariable logistic regression analysis was performed to investigate the health outcomes of adolescents who used smartphones for > 4 hours a day, compared to the control group. Statistical significance was determined using two-sided tests, with significance indicated by a *p* value < 0.05. The statistical analysis was performed using SAS version 9.4 (SAS Institute Inc., Cary, NC) using the KYRBWS weighted sampling design.

## Results

### Smartphone usage time of adolescents

Compared to the results of the KYRBWS conducted in 2017, adolescents in 2020 spent considerably more time on a smartphone on both weekdays and weekends. On a daily average, the proportion of adolescents using a smartphone for more than 2 hours a day increased from 64.3% in 2017 to 85.7% in 2020 (*p* < 0.001). In 2017, 35.7% of the population used smartphones for < 2 hours a day, which is the traditionally recommended usage time, but the proportion plummeted to 14.4% in 2020 (Table 1). In addition, 25.5% of adolescents experienced smartphone overdependence in 2020.

**Table 1:** Temporal trends of smartphone use in South Korean adolescent in youth risk behavior survey.

	2017 (N = 61,861)		2020 (N = 54,809)		
	N	Weighted %	N	Weighted %	*p* value
**Weekday**					< 0.001
< 2 h/day	31,481	51.3	12,689	23.1	
2–4 h/day	17,775	28.7	18,633	34.4	
> 4 h/day	12,605	20.0	23,487	42.5	
**Weekend**					< 0.001
< 2 h/day	18,173	29.9	6,914	13.2	
2–4 h/day	15,778	26.2	11,637	21.9	
> 4 h/day	27,910	44.0	36,258	64.9	
**Daily average**					< 0.001
< 2 h/day	21,873	35.7	7,688	14.4	
2–4 h/day	20,568	33.7	16,555	30.6	
> 4 h/day	19,420	30.6	30,566	55.1	
**Smartphone overdependence**			13,740	25.5	

Abbreviations: H, hours.

### Association between smartphone usage time and health outcomes

**Fig 2:**
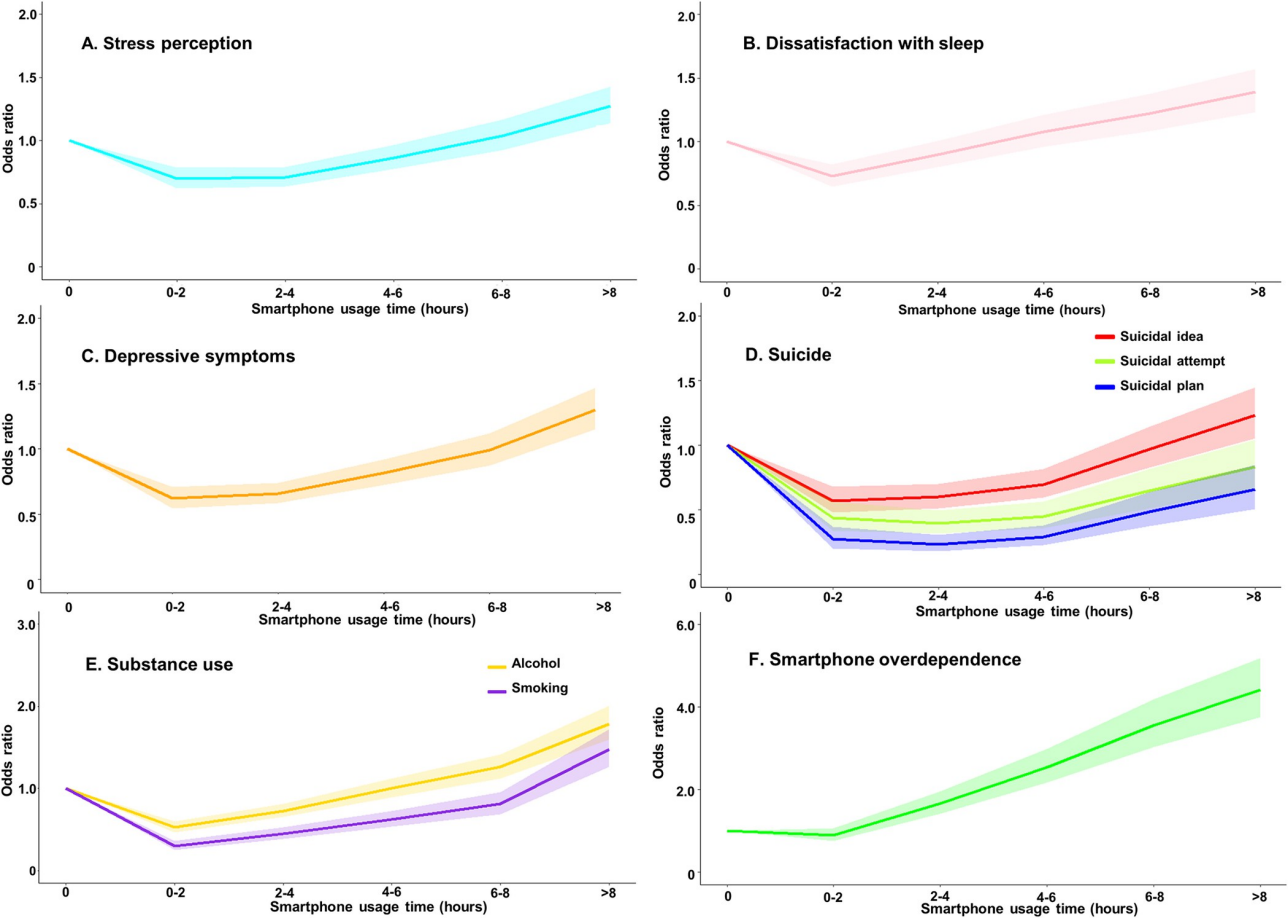
Association between daily smartphone usage time and health outcomes. The solid line represents the odds ratio. The x-axis represents the smartphone usage time group. The y-axis represents the odds ratio and non-user group is considered as a reference group. The shaded area represents the 95% confidence interval.

Fig 2 demonstrates the association between smartphone usage time and health outcomes. Compared to health outcomes for non-smartphone users, adolescents with < 2 hours of daily smartphone usage time had significantly lower odds ratios (OR) for stress perception (OR 0.70, 95% confidence interval [C.I]; 0.62–0.79), sleep dissatisfaction (0.73, 0.62–0.79), depressive symptoms (0.62, 0.55–0.71), suicidal ideation (0.57, 0.48–0.68), and substance use (alcohol, 0.53, 0.47–0.60) (S2 Table). In addition, these associations remained consistent in adolescents with 2–4 hours of usage time for health outcome variables including stress perception (0.71; 0.64–0.79), depressive symptoms (0.66, 0.59–0.74), suicidal ideation (0.60, 0.51–0.70), and substance use (alcohol, 0.73; 0.65–0.82). Most of the figures showed a curvilinear pattern in which the OR increased after 4–6 hours of usage time compared to that of non-users. Conversely, the association between smartphone usage time and smartphone overdependence was linear; overdependence and smartphone usage time increased in parallel. Finally, the association between obesity and smartphone usage time was not significant in every group.

### Comparisons of characteristics before and after PSM

The baseline demographic characteristics of the study population before and after PSM are presented in Table 2. Before matching, adolescents who spent more than 4 hours using smartphones were likely to be girls, with low socioeconomic status, and with low academic achievement. After matching, there were no differences in demographic characteristics between the > 4 hours a day group and < 4 hours a day group (SMD < 0.1).

**Table 2:** Description of demographic characteristics before and after propensity scoring matching.

	Before PSM					After PSM (1:1)				
	< 4 hours a day (N = 24,243)		> 4 hours a day (N = 30,566)		SMD	< 4 hours a day (N = 20,499)		> 4 hours a day (N = 20,499)		SMD
	N	Weighted %	N	Weighted %		N	Weighted %	N	Weighted %	
**Age**	15.07±0.03		15.28±0.03		-0.04	15.29±0.04		15.21±0.03		0.02
**Sex**										
Male	15,162	62.29	13,107	43.31	0.37	11,176	56.29	11,315	57.37	-0.02
Female	9,081	37.71	17,459	56.69	-0.37	8,608	43.71	8,469	42.63	0.02
**Residence**										
Large city	10,933	44.06	12,630	40.72	0.07	8,869	42.82	9,009	42.81	< 0.001
Medium-sized city	11,550	50.50	15,364	53.03	-0.05	10,098	51.75	9,943	51.95	< 0.001
Rural area	1,760	5.44	2,572	6.26	-0.03	1,532	5.43	1,547	5.23	< 0.001
**Socioeconomic status**										
High	10,796	45.64	10,475	35.14	0.21	8,350	42.51	8,216	40.31	0.04
Middle	10,936	44.52	15,421	50.06	-0.11	9,815	46.82	9,869	47.88	-0.02
Low	2,511	9.85	4,670	14.80	-0.15	2,334	10.68	2,414	11.81	-0.04
**Academic achievements**										
High	10,919	45.46	9,166	29.81	0.33	7,945	39.77	7,855	37.26	0.05
Middle	7,357	30.30	9,198	30.03	0.01	6,587	32.14	6,585	31.92	< 0.001
Low	5,967	24.23	12,202	40.16	-0.35	5,967	28.09	6,059	30.82	< 0.001

Abbreviations: PSM, propensity score matching; SMD, Standardized mean difference.

Data are expressed as number with weighted % in categorical variables and mean ± standard error in continuous variables.

## Effects of smartphone usage of more than 4 hours a day on health outcomes

**Fig 3:**
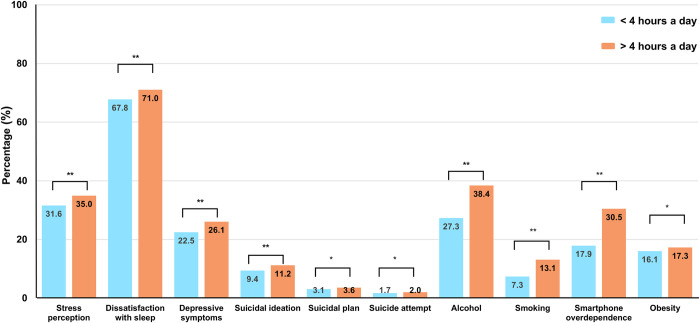
Comparisons of health outcomes between the groups of adolescents with < 4 hours of smartphone usage and > 4 hours of smartphone usage after propensity score matching. * p < 0.05, ** p < 0.001.

We compared the health outcome variables between the > 4 hours a day and < 4 hours a day groups (Fig 3). Compared to the health outcomes of individuals using a smartphone for < 4 hours a day, those using a smartphone > 4 hours a day had significantly higher rates of adverse mental health, prevalent substance use, and a higher rate of obesity (*p* < 0.001). Table 3 reflects the association between smartphones and health outcome variables before and after PSM. Among the mental health outcomes, adolescents using smartphones for > 4 hours a day were more likely to have a higher level of stress perception (adjusted OR; 1.16, 95% C.I. 1.11–1.22), dissatisfaction with sleep (1.17, 1.11–1.23), and depressive symptoms (1.22, 1.16–1.28) after PSM than those using a smartphone for < 4 hours. In addition, smartphone usage was significantly associated with all suicidal outcomes (suicidal ideation; 1.22, 1.13–1.31). Moreover, frequent smartphone usage was associated with prevalent substance use (alcohol; 1.66, 1.57–1.75), smartphone overdependence (2.01, 1.89–2.13), and obesity (1.09, 1.03–1.16). The overall OR values of outcome variables decreased but remained significant after PSM.

**Table 3:** Association between smartphone use and health outcome variables before and after PSM.

Health outcomes	Usage time	Before PSM			After PSM (1:1)		
		OR	95% C.I		OR	95% C.I	
**Stress perception**	> 4 h/day	1.38	1.33–1.44		1.16	1.11–1.22	
	< 4 h/day	1.00			1.00		
**Dissatisfaction with sleep**	> 4 h/day	1.39	1.34–1.45		1.17	1.11–1.23	
	< 4 h/day	1.00			1.00		
**Depressive symptoms**	> 4 h/day	1.43	1.37–1.50		1.22	1.16–1.28	
	< 4 h/day	1.00			1.00		
**Suicidal idea**	> 4 h/day	1.42	1.33–1.51		1.22	1.13–1.31	
	< 4 h/day	1.00			1.00		
**Suicidal plan**	> 4 h/day	1.35	1.22–1.49		1.17	1.04–1.31	
	< 4 h/day	1.00			1.00		
**Suicidal attempt**	> 4 h/day	1.46	1.27–1.68		1.20	1.03–1.40	
	< 4 h/day	1.00			1.00		
**Alcohol**	> 4 h/day	1.81	1.72–1.90		1.66	1.57–1.75	
	< 4 h/day	1.00			1.00		
**Smoking**	> 4 h/day	2.01	1.85–2.18		1.90	1.74–2.08	
	< 4 h/day	1.00			1.00		
**Smartphone overdependence**	> 4 h/day	2.29	2.18–2.41		2.01	1.89–2.13	
	< 4 h/day	1.00			1.00		
**Obesity**	> 4 h/day	1.00	0.94–1.05		1.09	1.03–1.16	
	< 4 h/day	1.00			1.00		

Abbreviations: PSM, propensity score matching; h, hours; OR, odds ratio; C.I, confidence interval.

## Discussion

Using a nationwide survey, we investigated the relationships between the smartphone usage and health outcomes of Korean adolescents. Our study demonstrated the curvilinear relationship between smartphone usage time and adverse health outcomes, with inflection points rather than a linear relationship. Excessive smartphone usage was related to adverse behavioral health outcomes, which were overt when the usage time exceeded 4 hours a day. This relationship could also be in the opposite direction, as previous studies have shown that adolescents with emotional regulation difficulties may be more prone to excessive smartphone use [25,26].

The increasing trend of smartphone usage in the 2010s, as stated in the literature, is likely ongoing and even accelerating [9]. According to a Canadian study, the COVID-19 outbreak led to increasing loneliness and depression in adolescents, and this phenomenon had a more prominent impact on those with high social media usage [27]. In addition, the outbreak accelerated the increasing availability of online education on mobile devices because of distance learning during the lockdown period.

PSU is defined as excessive smartphone usage with negative functional consequences. It has been measured in different ways by researchers, such as by using self-administered questionnaires based on the smartphone addiction scale [28,29] or usage time [9,13]. Furthermore, recent studies have investigated the association between smartphone usage and mental health by stratification according to the main purpose (e.g., online shopping, social media, games) [30,31]. However, we assumed that the primary purpose of smartphone use could not be limited to a solitary purpose, as smartphones have become the daily life platform of teenagers. The varying measurements of PSU reflect the lack of consensus regarding the definition.

We selected smartphone usage time as an independent variable, as it is the most objective way to measure smartphone usage. This study found curvilinear relationships between smartphone usage time and health outcomes. This implies that appropriate smartphone usage can be prosocial and enjoying smartphone use should not be conceptualized as a behavioral addiction such as gambling and playing video games. Children and teenagers spend considerable time using smart devices as a social platform for peer relationships (e.g., text messaging, social network services).

A study based on the 2017 KYRBWS showed that the more time adolescents spend on smartphones, the more time they utilize for social purposes. Regarding social purposes, 1–2 hours of usage time was protective against suicide attempts [13]. Thus, to some extent, smartphone usage can be considered in social and developmental contexts. Although individual inflection points were slightly different according to the outcomes of interest, using a smartphone for 4 hours a day seems to be generally acceptable. From our results, using smartphones for < 2 hours a day even seems beneficial for mental health outcomes compared to non-use. However, given the cross-sectional design of this study, the causality and long-term effects could not be demonstrated [19].

The curvilinear relationship shown in our study raises questions regarding the time setting of smart devices in adolescents. In concordance with the previous report, the result suggests that a daily limit of 2 hours may not be practical or realistic [17]. The AAP guideline recommended limiting digital media usage in both children and adolescents. In addition, parents have been advised to help children with safe social media use including setting a device turn-off time in the bedroom, at school, and 1 hour before bedtime [32]. Regarding children of preschool age, growing evidence suggests that early and prolonged exposures to digital media alter the microstructure of the brain’s white matter integrity, which can affect poor language and cognitive development [33]. However, previous guidelines or statements are not based on objective evidence regarding the time limit of smartphone use in adolescents. It has even been admitted that older children and teenagers spend up to > 11 hours per day using media [34]. As adolescents use smartphones not only for entertainment but also for other purposes including education and communication, a 2-hour limit similar to that for young children should be reconsidered.

Meanwhile, our study showed that adverse effects of health outcomes manifested after 4 hours of smartphone usage time, which corresponds with previous studies [16,17]. Caution must be taken in interpreting these results, which do not imply that using a smartphone for 4 hours a day is always undesirable. Evaluation of PSU based on smartphone usage time alone is not appropriate, and a more comprehensive understanding including age group, purpose of usage, and cultural differences is required [35]. Nevertheless, after controlling for several confounders that could affect health outcomes using PSM, our results suggest that smartphone usage beyond certain levels could significantly affect health outcomes. We further analyzed the data from the 2017 and 2020 KYRBWS separately, and the results are presented in S3 Table. Overall, smartphone usage > 4 hours per day adversely impacted mental health outcomes; however, the ORs for each variable showed a decreasing pattern, except for substance use. While still significant, we should take into account the characteristics of smartphone penetrance and universality, which could result in dynamic temporal relationship. We assume that the health-related effects of smartphones that most people worry about do not worsen over time.

Although our data did not include a wide range of behavioral outcomes, associations between smartphone usage time and adverse health outcomes were more prominent in externalizing behavioral difficulties (e.g., substance use) than internalizing behavioral difficulties (e.g., stress perception, depressive symptoms, suicide). Sarmiento et al. [36] showed that PSU was associated with internalizing symptoms, moderated by factors such as sex and peer relationships. According to a recent longitudinal study, an increase in smartphone usage over a year was more associated with externalizing behavior difficulties such as inattention and aggression than internalizing difficulties [37]. Smartphone use induces more usage time by satisfying the immediate reward needs of adolescents with externalizing behavior problems. Further longitudinal studies covering diverse behavioral outcomes can elucidate the detailed associations among them.

In our analysis, the relationships between smartphone usage and health outcomes became less prominent after PSM in regression analysis. It suggests that the environmental factors considered in PSM affect and strengthen the association. For example, previous studies showed that girls had a higher risk of smartphone addiction than boys [38,39]. We could not validate our assumption through the mediation study owing to the cross-sectional design with complex sampling.

To the best of our knowledge, this is the first study to address the association between smartphone usage time and various health outcomes in adolescents using a nationwide survey by employing PSM, which has the advantage of controlling for possible covariates. South Korea has the highest smartphone penetration rate, reaching as high as 95% in recent surveys [40]. The results of this study will provide useful information for establishing smartphone usage guidelines for adolescents and young adults by reflecting recent trends. However, our study was subject to several limitations. First, as our survey covered a cross-sectional database, causal relationships between smartphone usage and adverse health outcomes could not be confirmed. Second, we relied on individuals’ self-answered questionnaires on smartphone usage time and health outcomes. The reported usage time may not be an estimate of the actual usage time and could be underestimated due to the tendency to provide socially desirable and acceptable answers. Nevertheless, self-reported usage time in a nationwide representative survey has been widely used in similar studies since it reflects smartphone usage time in normal adolescent populations and comprehensively reflects a variety of purposes and situations that could be demanding in record-keeping or experimental designs [9,41–43]. Finally, we could not specify the smartphone usage time according to the purpose (e.g., social media use, text messaging, education, online shopping), which could have affected the health outcomes, owing to the innate limitation of our database.

In conclusion, our study revealed curvilinear relationships between smartphone usage time and undesirable health outcomes. The adverse effects of smartphone overuse became prominent after 4 hours of daily usage time. These results can help establish smart device usage guidelines and education programs for appropriate media use.

## Supporting information

S1 Table Questionnaires of Korean Youth Behavior study used in the study. (PDF)

S2 Table Logistic regression model presenting the association between smartphone usage time and health variable outcomes. (PDF)

S3 Table Regression model presenting the association between smartphone usage time and health variables outcomes in 2017, 2020 KYRBWS. (PDF)
