# Supplementary material for: Association between smartphone usage and health outcomes of adolescents: A propensity analysis using the Korea youth risk behavior survey
Source: PLoS One. 2023 Dec 6;18(12):e0294553. doi: 10.1371/journal.pone.0294553 (PMC10699629; doi:10.1371/journal.pone.0294553)
Supplement: S1 Table — (PDF) [file pone.0294553.s001.pdf]

Supplementary Table 1. Questionnaires of Korean Youth Behavior study used in the study

Questions	Answers	
**Category : Smartphone use**		
In the last 7 days, how many hours per day did you use the smartphone in average on weekdays?	Short answer with 10-minute increments (i.e., 10 minutes, 1 hour 40 minutes, etc.)	
In the last 7 days, how many hours per day did you use the smartphone in average on weekends?	Short answer with 10-minute increments (i.e., 10 minutes, 1 hour 40 minutes, etc.)	
**Category : Mental health**		
<Stress perception><br>How often you get stressed in daily life?	(1) very much<br>(2) a lot	Stressed
	(3) a little<br>(4) very little<br>(5) none at all	Unstressed
<Dissatisfaction with sleep><br>In the last 7 days, do you think you have had enough sleep?	(1) very much<br>(2) a lot	Good
	(3) a little<br>(4) very little<br>(5) none at all	Bad
<Depressive symptoms><br>In the past 12 months, have you felt any sadness and hopeless which made your daily life stop for 2 weeks?	(1) very much<br>(2) a lot	Yes
	(3) a little<br>(4) very little<br>(5) none at all	No
<Suicidal idea><br>In the past 12 months, have you seriously considered to suicide?	(1) very much<br>(2) a lot	Yes
	(3) a little<br>(4) very little<br>(5) none at all	No
<Suicidal plan><br>In the past 12 months, have you seriously planned to suicide?	(1) very much<br>(2) a lot	Yes
	(3) a little<br>(4) very little<br>(5) none at all	No
<Suicidal attempt><br>In the past 12 months, have you seriously tried to suicide?	(1) very much<br>(2) a lot	Yes
	(3) a little<br>(4) very little<br>(5) none at all	No
**Category : Substance use**		
<Alcohol><br>During the past 30 days, on how many days did you have at least one drink of any alcoholic beverage?	(1) none	No
	(2) 1 or 2 days<br>(3) 3 to 5 days<br>(4) 6 to 9 days<br>(5) 10 to 19 days<br>(6) 20 to 29 days<br>(7) every single day	Yes

(Continued)

<Smoking><br>During the past 30 days, on how many days did you have used a tobacco product?	(1) none	No
	(2) 1 or 2 days<br>(3) 3 to 5 days<br>(4) 6 to 9 days<br>(5) 10 to 19 days<br>(6) 20 to 29 days<br>(7) every single day	Yes
**Category : Obesity**<br><br>What is your recently measured height? (cm)<br><br>What is your recently measured weight? (kg)		
