# Supplementary material for: Association between smartphone usage and health outcomes of adolescents: A propensity analysis using the Korea youth risk behavior survey
Source: PLoS One. 2023 Dec 6;18(12):e0294553. doi: 10.1371/journal.pone.0294553 (PMC10699629; doi:10.1371/journal.pone.0294553)
Supplement: S2 Table — (PDF) [file pone.0294553.s002.pdf]

Supplementary Table 2. Logistic regression model presenting the association between smartphone usage time and health variable outcomes.

	Stress perception	Sleep dissatisfaction	Depressive symptoms	
Non-user	1.00	1.00	1.00	
0-2 h	0.70 (0.62-0.79)	0.73 (0.62-0.79)	0.62 (0.55-0.71)	
2-4 h	0.71 (0.64-0.79)	0.90 (0.80-1.00)	0.66 (0.59-0.74)	
4-6 h	0.87 (0.77-0.97)	1.08 (0.96-1.21)	0.82 (0.73-0.92)	
6-8 h	1.04 (0.93-1.17)	1.22 (1.08-1.38)	0.99 (0.88-1.12)	
> 8 h	1.28 (1.14-1.43)	1.39 (1.23-1.57)	1.30 (1.15-1.47)	
	**Suicidal idea**	**Suicidal plan**	**Suicidal attempt**	
Non-user	1.00	1.00	1.00	
0-2 h	0.57 (0.48-0.68)	0.44 (0.34-0.56)	0.27 (0.20-0.37)	
2-4 h	0.60 (0.51-0.70)	0.40 (0.32-0.50)	0.23 (0.18-0.30)	
4-6 h	0.70 (0.59-0.82)	0.45 (0.36-0.56)	0.29 (0.22-0.38)	
6-8 h	0.97 (0.82-1.14)	0.65 (0.51-0.82)	0.49 (0.37-0.63)	
> 8 h	1.23 (1.05-1.45)	0.83 (0.66-1.04)	0.66 (0.50-0.85)	
	**Alcohol**	**Smoking**	**Smartphone overdependence**	**Obesity**
Non-user	1.00	1.00	1.00	1.00
0-2 h	0.53 (0.47-0.60)	0.30 (0.25-0.36)	0.90 (0.76-1.07)	1.00 (0.87-1.17)
2-4 h	0.73 (0.65-0.82)	0.45 (0.39-0.53)	1.67 (1.42-1.96)	0.96 (0.83-1.10)
4-6 h	1.00 (0.89-1.11)	0.62 (0.53-0.73)	2.55 (2.17-2.98)	0.93 (0.80-1.07)
6-8 h	1.26 (1.12-1.42)	0.81 (0.69-0.95)	3.57 (3.04-4.20)	0.97 (0.83-1.12)
> 8 h	1.79 (1.59-2.00)	1.47 (1.26-1.72)	4.41 (3.76-5.18)	1.04 (0.89-1.20)

Abbreviations: h, hours;
Expressed as an odds ratio with 95% confidence interval.
