# Supplementary material for: Association between smartphone usage and health outcomes of adolescents: A propensity analysis using the Korea youth risk behavior survey
Source: PLoS One. 2023 Dec 6;18(12):e0294553. doi: 10.1371/journal.pone.0294553 (PMC10699629; doi:10.1371/journal.pone.0294553)
Supplement: S3 Table — (PDF) [file pone.0294553.s003.pdf]

**Supplementary Table 3. Regression model presenting the association between smartphone usage time and health variables outcomes in 2017, 2020 KYRBWS**

| Health outcomes | Usage time | 2017 | | 2020 | |
|---|---|---|---|---|---|
| | | OR | 95% C.I | OR | 95% C.I |
| **Stress perception** | > 4 h/day | 1.44 | 1.38-1.49 | 1.38 | 1.33-1.44 |
| | < 4 h/day | 1.00 | | 1.00 | |
| **Dissatisfaction with sleep** | > 4 h/day | 1.41 | 1.35-1.49 | 1.39 | 1.34-1.45 |
| | < 4 h/day | 1.00 | | 1.00 | |
| **Depressive symptoms** | > 4 h/day | 1.55 | 1.48-1.61 | 1.43 | 1.37-1.50 |
| | < 4 h/day | 1.00 | | 1.00 | |
| **Suicidal idea** | > 4 h/day | 1.58 | 1.50-1.67 | 1.42 | 1.33-1.51 |
| | < 4 h/day | 1.00 | | 1.00 | |
| **Suicidal plan** | > 4 h/day | 1.47 | 1.34-1.61 | 1.35 | 1.22-1.49 |
| | < 4 h/day | 1.00 | | 1.00 | |
| **Suicidal attempt** | > 4 h/day | 1.77 | 1.57-1.99 | 1.46 | 1.27-1.68 |
| | < 4 h/day | 1.00 | | 1.00 | |
| **Alcohol** | > 4 h/day | 1.77 | 1.69-1.85 | 1.81 | 1.72-1.90 |
| | < 4 h/day | 1.00 | | 1.00 | |
| **Smoking** | > 4 h/day | 1.72 | 1.61-1.82 | 2.01 | 1.85-2.18 |
| | < 4 h/day | 1.00 | | 1.00 | |
| **Smartphone overdependence *** | > 4 h/day | | | | |
| | < 4 h/day | | | 1.00 | |
| **Obesity** | > 4 h/day | | | 2.29 | 2.18-2.41 |
| | < 4 h/day | 1.07 | 1.01-1.13 | 1.00 | 0.94-1.05 |

Abbreviations: KYRBWS, Korean Youth Risk Behavior Web-based Survey; h, hours; OR, odds ratio; C.I, confidence interval.
* Question regarding smartphone overdependence was performed since 2020.
